# From novice to expert: methods for transferring implementation facilitation skills to improve healthcare delivery

**DOI:** 10.1186/s43058-021-00138-5

**Published:** 2021-04-08

**Authors:** Mona J. Ritchie, Louise E. Parker, JoAnn E. Kirchner

**Affiliations:** 1 VA Behavioral Health Quality Enhancement Research Initiative (QUERI), Department of Veterans Affairs, 2200 Ft Roots Dr, Building 58, North Little Rock, AR 72114 USA; 2 Department of Psychiatry, University of Arkansas for Medical Sciences, 4301 W Markham St, #755, Little Rock, AR 72205 USA; 3 Department of Management, College of Management, University of Massachusetts, 100 Morrissey Blvd, Boston, MA 02125 USA

**Keywords:** Facilitation, Implementation, Skill transfer, Implementation strategy

## Abstract

**Background:**

There is substantial evidence that facilitation can address the challenges of implementing evidence-based innovations. However, facilitators need a wide variety of complex skills; lack of these can have a negative effect on implementation outcomes. Literature suggests that novice and less experienced facilitators need ongoing support from experts to develop these skills. Yet, no studies have investigated the transfer process. During a test of a facilitation strategy applied at 8 VA primary care clinics, we explored the techniques and processes an expert external facilitator utilized to transfer her skills to two initially novice internal facilitators who became experts.

**Methods:**

In this qualitative descriptive study, we conducted monthly debriefings with three facilitators over a 30-month period and documented these in detailed notes. Debriefings with the expert facilitator focused on how she trained and mentored facilitation trainees. We also conducted, recorded, and transcribed two semi-structured qualitative interviews with each facilitator and queried them about training content and process. We used a mix of inductive and deductive approaches to analyze data; our analysis was informed by a review of mentoring, coaching, and cognitive apprenticeship literature. We also used a case comparison approach to explore how the expert tailored her efforts.

**Results:**

The expert utilized 21 techniques to transfer implementation facilitation skills. Techniques included both active (providing information, modeling, and coaching) and participatory ones. She also used techniques to support learning, i.e., cognitive supports (making thinking visible, using heuristics, sharing experiences), psychosocial supports, strategies to promote self-learning, and structural supports. Additionally, she transferred responsibility for facilitation through a dynamic process of interaction with trainees and site stakeholders. Finally, the expert varied the level of focus on particular skills to tailor her efforts to trainee and local context.

**Conclusions:**

This study viewed the journey from novice to expert facilitator through the lens of the expert who transferred facilitation skills to support implementation of an evidence-based program. It identified techniques and processes that may foster transfer of these skills and build organizational capacity for future implementation efforts. As the first study to document the implementation facilitation skills transfer process, findings have research and practical implications.

**Supplementary Information:**

The online version contains supplementary material available at 10.1186/s43058-021-00138-5.

Contributions to the literature

- This study fills a gap in the literature about how experts can help novice and less experienced facilitators develop the complex skills needed for facilitating implementation of evidence-based innovations.
- We identify and describe techniques, processes, and patterns of interaction that an expert facilitator utilized to transfer implementation facilitation skills to initially novice facilitators, enabling them to become experts.
- Our findings can inform the transfer of skills needed to apply facilitation, and potentially other implementation strategies, to healthcare system change agents, thereby building capacity for implementing evidence-based practices, programs, and policies.

## Background

Implementation experts propose that facilitation is a necessary component for successful implementation [1]. Additionally, there is substantial evidence that facilitation can indeed help to successfully address the challenges of implementing evidence-based practices, programs, and policies and improve healthcare delivery [2–7]. Although some organizations have natural internal facilitators who support implementation of innovations, many healthcare systems lack infrastructure support, resources, and/or knowledge to support organizational change [8–10]. Thus, some studies and clinical initiatives enlist an expert facilitator, external to the organization needing assistance, to help internal stakeholders implement an innovation [11]. Having expert facilitators who are able to transfer implementation facilitation skills to internal change agents may foster healthcare systems’ ability to build capacity for implementing evidence-based innovations [3, 4]. This article focuses on how an expert can transfer facilitation skills and provide support to novice and less experienced facilitators.

Implementation facilitation is a “multi-faceted interactive process of problem solving, enabling and supporting individuals, groups, and organizations in their efforts to adopt and incorporate innovations into routine practices that occurs in the context of a recognized need for improvement and a supportive interpersonal relationship” [12]. Empirical research and theory have documented a multitude of factors that influence implementation [9, 13–16], and facilitators need to be able to identify and address these by selecting and applying multiple discrete implementation strategies [12, 17] based on the needs and resources of the organizational context. The integrated-Promoting Action on Research Implementation in Health Services (i-PARIHS) framework posits that facilitation is the *active* ingredient in the implementation process; facilitators need to assess and respond to the characteristics of the innovation being implemented, the individuals and teams involved in or affected by innovation implementation, and the organizational context [1, 18]. Thus, facilitators need a wide range of very complex skills [19]. Lack of these skills can compromise fidelity to the facilitation intervention and ultimately has a negative effect on outcomes [20–23]. Implementation initiatives applying facilitation need to ensure that facilitators have the necessary skills or provide training to develop them [1, 24]. Therefore, understanding how to transfer these complex skills is vitally important.

Facilitators and others in similar roles, e.g., knowledge brokers [25], learn about how to support implementation through formal training programs that use didactics [26, 27] and/or participatory methods [28–31] in short, time-limited workshops [32] or multiple workshops [26, 29–31, 33]; on-the-job training; ongoing mentoring [3]; or a combination of workshops(s) and mentoring [26, 30, 34]. They may learn through publicly available materials [26, 35, 36] and/or through experience by trial and error [37]. Learning about implementation facilitation and the activities facilitators perform is necessary but not sufficient for developing these complex skills. First, similar to the skills needed for the practice of medicine, nursing, and teaching, facilitation skills include both explicit and tacit knowledge. We can use words and symbols to express and document explicit knowledge; thus, didactic instruction and/or training materials may be useful mechanisms for transferring it [38]. Tacit knowledge takes the form of beliefs, understandings, skills, and practices [39]; it is generally acquired through experience [40] and by observing and working with someone who has expertise. Although some tacit knowledge can be communicated verbally, workshops and training materials are not sufficient for helping others learn new complex skills that include tacit dimensions [38, 41]. Second, applying new complex knowledge and skills is challenging because newly learned behavior is crude compared to that of an expert, is fragile in the face of the reactions of others, and is incomplete when applied in the setting in which it will be used [42].

Some scholars propose that non-expert facilitators need ongoing support from expert or more experienced facilitators to develop their skills [1, 43, 44]. Developers of the i-PARIHS framework even proposed a pathway from novice to expert facilitator that includes ongoing mentoring and support [1, 18]; they did not, however, identify techniques and processes that experts can use to provide such support. A scoping review that sought to identify interventions and strategies for teaching and reinforcing core knowledge translation competencies found few mentioned in the literature and concluded that further research was needed [45]; and recent evaluations of knowledge translation competency trainings focus on the techniques used in group-based training rather than in ongoing mentoring and support processes [30, 31]. Scholars have described techniques and processes for transferring complex skills in other fields, e.g., medicine and nursing [46–49], and often, experts who serve as supervisors in these fields receive specific training in skills transfer [50, 51]. Given the effectiveness and increasing use of implementation facilitation, the complexity of skills, and the importance of facilitators having the appropriate skills, we need to understand how experts and more experienced facilitators can help others learn how to support implementation. This is the first study to explore methods and techniques experts can use to transfer implementation facilitation skills.

We conducted this study within the context of a large Department of Veterans Affairs (VA) funded project that applied a facilitation strategy to support implementation of evidence-based primary care mental health integration (PCMHI) care models in eight VA primary care clinics. The original PARIHS framework informed the design of our facilitation strategy, a blend of external and internal facilitation [37, 52]. We tested the strategy by comparing outcomes to eight clinics that did not receive facilitation [3, 4]. The project was an enhancement to a VA initiative to improve PCMHI implementation that included a mandate and national level resources, i.e., consultation, technical assistance, education and training, and informational tools [53]. By design, in addition to supporting implementation, the expert external facilitator transferred facilitation skills to healthcare system change agents, who were initially novices but became expert facilitators by the end of the project. A previous study documented the complex skills the expert transferred [19]; this study, also a component of the larger project and the first author’s doctoral dissertation [54], explored how the expert transferred these skills to support implementation of PCMHI, as well as build capacity for future implementation efforts. For this paper, we call the internal facilitators, facilitation trainees.

## Methods

We adopted a qualitative descriptive study design [55, 56] to inform our exploration of the skills transfer process. Such methods focus on conducting in-depth exploration and discovery of new information that can form the basis of quantitative measures and testable hypothesis for future rather than confirmation of existing theory. It is by nature very labor intensive in both the data collection and analysis phases, consequently very expensive, and thus must scarify breadth (i.e., large sample sizes) for depth (i.e., deep study of a small sample) [57].

### Study setting

Eight primary care clinics, four in each of two VA regional networks, receiving facilitation for the larger project, provided the setting for this study. We selected VA networks based on (1) ability to identify a facilitation trainee who would support PCMHI implementation at fifty percent effort and (2) willingness to participate in the study. Mental health leaders in each of the two networks then identified one VA medical center and three community-based outpatient clinics of varying sizes where the primary care clinics planned to implement PCMHI in the first year of the study but would have difficulty without implementation assistance. More thorough descriptions of the study setting have been previously published [3, 4].

### The implementation facilitation intervention

An external facilitator, JEK, with expertise in PCMHI care models, implementation science, facilitation, and mentoring and one facilitation trainee in each of the two VA networks applied the strategy. Facilitation trainees, a doctoral level psychologist and a master level social worker, were network level employees who had no implementation science or facilitation expertise. Network mental health leaders identified staff for the facilitation trainee roles, and the expert facilitator worked with the trainees to transfer implementation facilitation skills from May and July 2009 until November 2011. Across this time period, facilitators helped primary care clinics and their parent VA medical centers implement PCMHI. Descriptions of their activities have been well-documented elsewhere [3, 4, 58]. Transferring implementation facilitation skills to facilitation trainees so that over time they also became experts was a key component of the expert’s role.

### Data collection

For the larger project, we debriefed the expert and two initially novice facilitators, took notes at pre-site visit facilitator meetings, and conducted semi-structured qualitative interviews with all three facilitators. Data collected served as source data for this study. We conducted joint debriefings with the expert and relevant facilitation trainee immediately after the initial visit to each site and conducted monthly individual, approximately hour long, debriefings with all three facilitators from August 2009 to November 2011. During expert facilitator debriefings, we focused primarily on documenting her efforts to help trainees learn how to support implementation. During facilitation trainee debriefings, we documented the facilitation process and implementation progress at each site, and we collected information relevant to the expert facilitator’s training. In total, we conducted 85-h-long debriefings by telephone, taking extensive notes, and documenting facilitators’ responses as close to verbatim as possible. After one interviewer drafted the notes, both reviewed and came to consensus on their content.

We also conducted semi-structured qualitative interviews by telephone with each of the facilitators to assess the implementation facilitation and skills transfer processes approximately 16 months after the initial site visit and again at the end of the study. These 1- to 2-h qualitative interviews sought to assess their perceptions of the skills transfer processes, what was particularly salient for each of the facilitators, and how the external facilitator worked with each of the facilitation trainees. In total, we conducted six interviews with facilitators, which we audio-recorded, and produced verbatim transcripts.

Two highly experienced female qualitative researchers conducted all data collection activities. The primary interviewer (LEP) is an organizational scientist and health policy and management expert, and the second interviewer (MJR) earned a PhD in Public Policy during the study. Both interviewers had personal relationships with the expert (JEK). The VA Central Institutional Review Board (#09-05) approved the conduct of the larger project, including the documentation of facilitator’s quality improvement activities in debriefings and meeting notes. We conducted a verbal consent process with the two facilitation trainees prior to conducting the semi-structured qualitative interviews described above.

### Data analysis

We used a mix of inductive and deductive approaches to conduct a content analysis [59] of the source data to explore techniques the expert applied and patterns of interaction that supported the skills transfer process. We then used a case comparison approach to explore how the expert tailored her efforts to trainee and context.

To identify skills transfer techniques, we developed a codebook and coded source material. Although to date no studies have explored what methods and techniques expert facilitators use to support the transfer of their knowledge and skills, there is information about skills transfer techniques from other fields such as medicine, nursing, and teaching, as well as career development [49, 60–63]. Thus, MJR conducted a search of mentoring, coaching, and cognitive apprenticeship literature across a wide range of fields from 1983 to 2014 in seven databases (CINAHL Plus, ERIC, Google Scholar, OVID, PubMed, SocINDEX, and Web of Science) and also searched for books on these topics. She identified and reviewed 79 journal articles, 10 books, and 5 book chapters to compile a list of methods and techniques utilized for transferring complex skills. This list (See Additional file 1) formed the foundation for the initial codebook for this study. Additionally, the codebook included codes to capture information about the facilitation trainees, the organizational context, and methods the expert used to tailor her efforts. Using ATLAS.ti (2016), MJR applied the initial codebook to the source data and refined the codes and definitions throughout the analysis process. She coded “instances” in which the expert was helping trainees learn a particular skill only once. If the expert mentioned an instance more than once or if it was clear that text was referring to an instance more than once, she applied all relevant codes to the first mention of this instance and hyperlinked related text to the first instance. Additionally, because facilitation trainee debriefing notes provided a rich source of background information, MJR hyperlinked relevant text in trainee debriefing notes to text in the expert’s debriefing notes. She then conducted an in-depth exploration of coded text to understand and describe the techniques and inductively clustered these into categories ([64], p. 275). Finally, she developed descriptions of the skills transfer techniques and processes the expert used.

To explore patterns in the data, MJR created Excel spreadsheet data displays for each skill [19] that the expert transferred, documenting, for each instance, the expert’s technique(s); date; trainee; site(s); primary target of the expert’s efforts (e.g., trainee, site leader, meeting attendees); and an abbreviated summary of what the expert was helping trainees learn. Using these data displays, she explored patterns in how facilitators were interacting with each other and with site stakeholders, as well as changes in the level of intensity with which interactions occurred over time.

Finally, MJR utilized a case comparison approach to explore whether/how the expert tailored her level of focus on particular skills to facilitation trainees and organizational context. For each of the skills we identified in a previous study [19], she assessed variation in the number of instances in which the expert helped trainees, assuming that the number of instances indicated the expert’s level of focus on that skill. To create level of focus ratings, we clustered the data into five categories (Low, Low Moderate, Moderate, High Moderate, and High; see Table 1). To identify variation, MJR then compared trainees on level of focus ratings and how level of focus changed over time. When there was variation between trainees, either overall or over time, she explored the data for ways in which the expert was tailoring her level of focus to trainee or context. The external facilitator, JEK, and one of the facilitation trainees reviewed and confirmed all findings.

**Table 1:** Cut-points for level of focus ratings

Cut-points	Ratings
≥ 20 instances	High
17–19 instances	High moderate
13–16 instances	Moderate
10–12 instances	Low moderate
0–9 instances	Low

## Results

### Techniques and processes for transferring skills

**Fig. 1:**
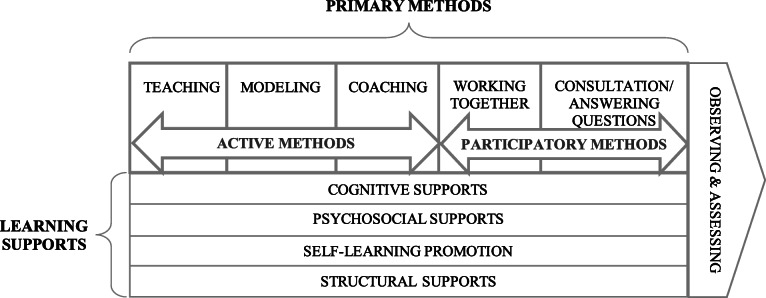
Types of techniques and processes for transferring implementation facilitation skills. Legend: Primary methods for directly transferring implementation facilitation knowledge and skills include both active and participatory methods/techniques. Learning supports promote the process of developing these skills. Throughout the process of transferring skills, the expert observed and assessed trainees to identify what they needed and which techniques and processes would support the transfer of skills

We identified 21 techniques the expert facilitator utilized to transfer implementation facilitation skills. The expert applied one of these techniques, observation and assessment of trainees’ efforts, throughout the transfer process to inform decisions about what content to provide and which techniques she should use. We clustered the remaining 20 techniques, which the expert used to directly support trainees’ learning, into 2 broad categories: (1) primary methods and (2) learning supports. Figure 1 presents a model of the types of techniques and processes the expert utilized, and below, we describe these categories and techniques.

### Primary methods

Primary methods consisted of *active methods*, i.e., those the expert initiated and utilized to directly transfer skills to trainees, and *participatory methods*, those she used in response to trainee requests or when they were conducting facilitation activities together. *Active methods* included teaching, modeling, and coaching. She used teaching to provide new information about PCMHI and the process of implementation facilitation. She performed facilitation activities while trainees watched, thus modeling how to facilitate. She also coached trainees before, during, and/or after facilitation efforts by providing suggestions and advice on how to conduct facilitation activities. *Participatory methods*, including working together and providing consultation, were generally used when trainees no longer needed such direct methods to continue to build their skills. When the expert and trainees were collaboratively conducting facilitation activities, the expert provided information or coaching as needed. She also provided this assistance when trainees were working independently and requested it. The expert primarily used active methods at the beginning of the first year, more participatory methods toward the end of that year, and predominantly participatory methods throughout the second year. It is important to note that she used both active and participatory methods, as well as many of the learning supports described below, across the entire process of working with facilitation trainees. Table 2 provides information about when the expert used primary methods and what was salient about these methods for trainees.

**Table 2:** Primary methods, when the expert utilized them, and what was salient for trainees

	Timing	What was salient for facilitation trainees
**Active methods**		
Teaching	Mostly in the first 3 months	“It would have been very helpful to me to have had a much more intensive...knowledge and information from the very, very beginning of the process.”
Modeling	Throughout the process but most frequently from 2 to 8 months after she began working with them	In addition to modeling implementation facilitation activities, one trainee said, “So I have seen from her the modeling of how to be, I think, a very efficient, high powered but yet person centered and warm leader.”
Coaching	In the beginning for some of the less complex tasks. For more complex tasks, she used modeling first and later coaching, both prospectively and retrospectively	“I think by her sort of coaching… well this is how I would approach it, or this is what we need to do and sort of learning how to navigate within those systems but yet not coming across as critical, but coming across as being more helpful, to influence change.”
**Participatory methods**		
Working together	During the first year, the expert worked with trainees on facilitating less-complex processes. Their work was more collaborative generally after the first year.	“It has also switched…to…more of a collaborative, we’re working on this and less of a, I am mentoring you through this.”
Providing consultation	As trainees developed their skills, they conducted more activities independently but consulted with the expert as needed. The expert spoke little about this process.	Both trainees felt that being able to obtain consultation was one of the most important aspects of the expert’s work with them. One said, “….having somebody that is knowledgeable…if I get stumped…I can call.”

### Learning supports

In addition to the primary methods for transferring skills, the expert used four types of techniques to support trainees’ learning. She used *cognitive supports* (sharing experiences and telling stories, making her thinking visible, comparing facilitation to clinical skills and activities, and using heuristics or rules of thumb) to help trainees understand, apply, and generalize implementation facilitation processes. She used *psychosocial supports* (providing acceptance, confirmation, and support; providing protection; facilitating exposure/visibility and promoting trainee interests) to build and enhance trainees’ sense of competence, identity as facilitators, and effectiveness. To support trainees’ assessment, planning, and learning skills, the expert *promoted self-learning* by encouraging articulation and learning from others. The expert also used *structural supports* to provide trainees with opportunities to learn skills, including scheduling learning opportunities, using teachable moments, encouraging and empowering trainees to take on new roles, stepping in and stepping out, and pulling back and fading. Table 3 describes how the expert applied specific techniques the expert used in each category.

**Table 3:** Learning support techniques

**Cognitive learning supports**	
*Sharing experiences and telling stories* Providing trainees with examples of previous experiences, including stories about how other sites had addressed challenges or adapted PCMHI to their local context and using this technique with stakeholders to model the power it has for transferring knowledge	
*Making thinking visible* Explaining why she had acted with or responded to stakeholders in a particular way, suggesting this would help them learn how to facilitate in those circumstances and generalize learning to similar situations.	
*Using comparisons to clinical skills and activities* Comparing the process of assessing and addressing problems, e.g., destructive interpersonal and organizational dynamics, to clinical processes, e.g., she suggested that the process of facilitating a chaotic meeting was “*similar to doing a treatment group*”	
*Using heuristics or rules of thumb* Sharing rules of thumb to provide trainees with generalizable lessons on how to help sites. For example, “You get dealt the cards; make it into the best hand you can” (for dealing with challenges over which they had no control); “work with sites where they are” (for when stakeholders insisted on discussing local issues rather than focusing on the planned agenda); and “don’t plow ahead with your plan” (for when something, e.g., a prepared presentation, was not working)	
**Psychosocial learning supports**	
*Acceptance, confirmation, and support.* Praising trainees when they had good ideas or applied facilitation appropriately, providing confirmation when they accurately diagnosed problems, supporting their perceptions of what was happening, and providing ongoing support for the learning process	
*Providing protection.* Protecting trainees from making mistakes by conducting facilitation activities until they were ready or stepping in and taking over, e.g., leading a meeting, when trainees did not know how or were not ready to handle complex problems	
*Facilitating exposure and visibility and promoting trainee interests.* Calling attention to the role of trainees and deferring to them to ensure that they were seen as “the face of the program” and credible	
**Promoting self-learning**	
*Encouraging articulation.* Asking questions to encourage them to think aloud to, for example, prepare for meetings (what they planned to accomplish, what obstacles they might encounter, and how they might address these) or process what occurred during a meeting (what they thought about the meeting, who “key allies” might be, potential problems or barriers to implementation, potential next steps)	
*Encouraging learning from others.* Encouraging trainees to learn from other experts (e.g., by arranging meetings or referring them for consultation) and from similar others (e.g., to obtain materials or learn about what was working for them)	
**Structural learning supports**	
*Setting up opportunities.* Scheduling meetings with them on a regular or less frequent basis as needed	
*Using teachable moments*. Taking advantage of “teachable moments,” e.g., to help one of the trainees modify her interpersonal style to be more supportive, the expert took advantage of multiple opportunities to model and coach the trainee on how to more positively reinforce site efforts rather than point out their weaknesses	
*Encouraging and empowering to take on new roles.* Initially charging trainees with gathering information about sites and current practices while the expert conducted most facilitation activities. Within several months, encouraging, empowering, and sometimes “pushing” trainees to take on other new roles, e.g., leading meetings with site leadership and staff, as well as becoming the expert on site and network interpersonal and political dynamics and how to assess and monitor them	
*Stepping in and stepping out.* Stepping in (e.g., to say something or assume leadership) and stepping out (e.g., to hand leadership back to the trainee) as a way of protecting facilitation trainees from making mistakes or reinforcing other lessons they were learning; the expert suggested that this process involved “….*knowing when you get out of the way and just hold your breath…versus when you continue to kind of keep your hands on and be there standing in the corner to step in if you need to*”	
*Pulling back and fading.* Becoming “*increasingly less involved*” over time and stepping back and watching trainees; one of the trainees said, *“.…instead of her handling it, she would sort of advise me how to handle it”*	

### Patterns of interaction supported skills transfer

**Fig. 2:**
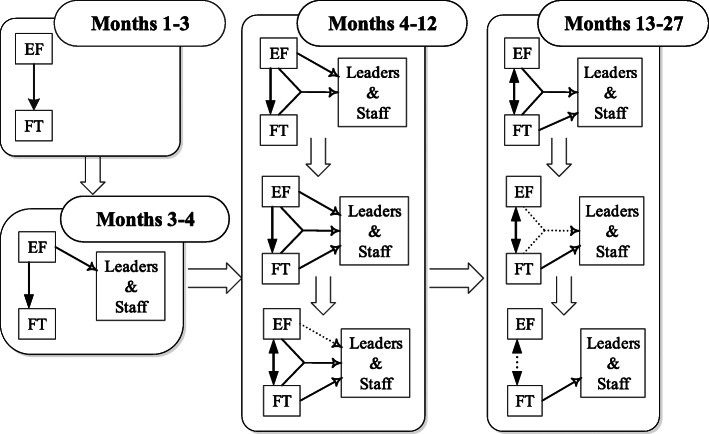
Patterns of interaction that supported the skills transfer process. Legend: Dark arrows indicate interactions between the expert and facilitation trainees with unidirectional arrows indicating that the expert was predominantly using active methods to transfer skills and the bidirectional arrows indicating she was using predominantly participatory ones. Lighter arrows depict facilitation activities with stakeholders and which of the facilitators was performing these activities. Dashed lines indicate that the types of interactions occurred infrequently. *EF* expert facilitator *FT* facilitation trainee

In addition to identifying techniques, we found patterns to the expert’s interactions with facilitation trainees and site stakeholders. In months 1–3, the expert worked with trainees and network mental health leaders to prepare for visiting local sites; facilitation trainees interacted minimally with site stakeholders for purposes of gathering information about the context and current PCMHI practices, as well as scheduling site visits. From 3 to 4 months, both facilitators interacted with site stakeholders, but the expert led all meetings and calls. From 4 to 12 months, initially, the expert led facilitation activities; but at times, she and the trainee interacted with site stakeholders together. By the end of this period, trainees were conducting many facilitation activities independently, and the expert was increasingly handing off the leadership role to them. By the end of the first year, she felt that trainees were able to facilitate implementation independently but continued to provide consultation as needed. Figure 2 illustrates the patterns of interaction between facilitators and between facilitators and stakeholders.

### Tailoring to needs, characteristics, and context

Our exploration of how the expert tailored her efforts to facilitation trainees and organizational context revealed that the expert initially assessed facilitation trainees’ strengths, weaknesses, and base knowledge; used a “shadowing process” to monitor them over time; and tailored her efforts to meet their particular needs. In addition to descriptions of how she accomplished the latter, comparison between ratings of her level of focus on specific skills provided us with additional information. The expert’s level of focus on specific skills varied across trainees for some skills (see Table 4). For example, the level of focus ratings on skills for “Interacting and working with leaders” was Low for Trainee-A and High for Trainee-C; and ratings for “Leading and managing team processes” were High Moderate for Trainee-A and Low for Trainee-C. On the other hand, the level of focus ratings was the same across facilitation trainees for other skills. For example, for both trainees, the ratings on “Engaging stakeholders” and “Problem-identification/solving skills” were High and ratings on skills for “Presenting and using data to improve the program” and “Integrating the program into the system” were Low. Overall, we found that the expert focused more on helping Trainee-A learn facilitation skills compared to Trainee-C. It may be that Trainee-C did not feel that she needed as much assistance. According to the expert facilitator, Trainee-C “broke away more cleanly than did [Trainee-A] and started doing things that I would not even be CCed on and then I would find out whenever we would have a meeting.” During the second year, the expert did little training and mentoring with Trainee-C except during one 3-month period in which she helped Trainee-C address some particularly difficult problems at study sites.

**Table 4:** Expert facilitator level of focus on individual skills by facilitation trainee

Implementation facilitation skills^1^	Trainee A	Trainee C
Engaging stakeholders	High	High
Problem-identification/solving	High	High
Learning from experts/similar others/experience	High	High
Communication skills	High	High Moderate
Interpersonal skills	High	High Moderate
Interacting and working with leaders	Low	High
Establishing learning collaboratives	High moderate	High moderate
Education and marketing skills	High moderate	Moderate
Leading and managing team processes	High moderate	Low
Assessment skills	Moderate	Moderate
Helping to design adapt a program to meet local needs	Moderate	Low
Developing a program monitoring system	Moderate	Low Moderate
Motivating and building confidence	Low	Moderate
Administrative and project management skills	Low moderate	Moderate
Training/mentoring and coaching	Low moderate	Low
Thinking strategically and planning	Low moderate	Low
Political skills	Low	Low Moderate
Presenting and using data to improve the program	Low	Low
Integrating the program into the system	Low	Low
Pulling back and disengaging	Low	Low
Meeting facilities and individuals where they are	Low	Low
Monitoring implementation	Low	Low

1[19]

Number of instances per rating: Low = 0–9; Low Moderate = 10–12; Moderate = 13–16; High Moderate = 17–19; High ≥ 20

Debriefing interviews provided us with information about how and why the expert tailored the level of focus on particular skills to trainees. For example, she told us that Trainee-A was an adept mental health provider and therapist; she targeted Trainee-A’s clinical skills and “translated” them to organizations by comparing facilitation activities to clinical assessment and intervention. The expert said that she encouraged her to actively listen during meetings and suggested that “when there is a real tense, chaotic environment … letting everyone have a voice.” We found that the expert tailored the skills transfer process to the past experiences, characteristics, and skills facilitation trainees had or lacked. She also tailored her efforts to organizational context to enable them to respond to site and network needs. Table 5 provides some additional examples of how and why the expert tailored the skills transfer process.

**Table 5:** Examples of how and why the expert facilitator tailored the skills transfer process

How the expert tailored her efforts	Why the expert may have tailored her efforts
Focused on helping Trainee-A develop leading and managing team process skills at a High Moderate level (compared to Low level with Trainee-C, who had this skillset); modeled how to lead/manage team processes; coached Trainee-A before, during and after meetings; led meetings when Trainee-A was not ready; stepped in and out when she needed help, and encouraged her to take on this new role.	− Trainee-A had little experience leading task-oriented meetings. − Trainee-A’s interpersonal style was thoughtful and laid back; she was inclined to be indecisive in meetings with stakeholders. − Site leaders and staff had strong personalities and expressed opinions forcefully.
Focused on helping Trainee-A learn training, mentoring, and coaching skills at a Low Moderate level (compared to Low level with Trainee-C); coached Trainee-A on using an enforcer role and enlisted Trainee-C to help Trainee-A learn the needed skills.	− Trainee-A was inclined to use a gentle coaching style with PCMHI providers. − The expert was concerned that this style was unlikely to motivate providers resistant to change or struggling with changing from traditional mental health to PCMHI.
Focused on helping Trainee-A learn education and marketing skills at a High Moderate level (compared to Moderate with Trainee-C).	− Network A had existing infrastructure support for a model of PCMHI that was not compliant with national requirements. − Network and clinic leaders and providers lacked perceived need to change.
Focused more in the first year on helping Trainee-A learn facilitation skills compared to Trainee-C and considerably more in the second year.	− Trainee-A was young, early in her career, less confident, and tended to rely on the expert even when she no longer needed the her. − Trainee-C became comfortable with her new role and began acting independently far sooner than Trainee-A.
Targeted Trainee-A’s clinical skills and “translated” them to organizations by comparing facilitation activities to clinical assessment and intervention.	− Trainee-A was an adept mental health provider and therapist.
Supported Trainee-C’s “natural aptitude” for working at the system level; viewed this style as valuable though different from her own.	− Trainee-C had clinical training and extensive experience in program QI and system redesign efforts; she was inclined to address system level issues, e.g., she focused more than Trainee-A on developing trainings and conferences. − Network C lacked infrastructure support for PCMHI.
Focused on helping Trainee-C learn (1) how to interact and work with leaders at a High level compared to focusing at a Low level with Trainee-A; (2) political skills at a Low Moderate level with Trainee-C and a Low level with Trainee-A; (3) how to engage leaders, assume a leadership role, provide advice and consultation, and interact with leaders comfortably.	− Trainee-C had previous experience in program management and quality improvement but within hierarchical systems under the operational authority of leaders and thus was inclined to defer to them. − Trainee-C had difficulty engaging VAMC leaders to support implementation.
Focused on helping to motivate stakeholders and build their confidence at a Moderate level with Trainee-C and at a Low level with Trainee-A. She worked with Trainee-C to develop a more positive attitude toward stakeholders and modeled how to interact with them from a strength perspective, e.g., by praising them for what they were able to accomplish.	− Trainee-C’s communication style was direct and somewhat abrupt; she tended to focus on negatives when providing feedback to stakeholders on implementation progress. − The expert was concerned that Trainee-C’s interpersonal style could be a barrier to engaging stakeholders and fostering PCMHI adoption.

## Discussion

Facilitation, a multi-faceted implementation strategy, is effective in improving uptake of evidence-based innovations. Without the appropriate skills, facilitators may have difficulty supporting implementation, which ultimately could affect both implementation and outcomes. Standalone training, even with interactive components, will likely not be sufficient for transferring such complex skills. Rather, scholars have suggested that ongoing mentoring and support is needed for effective skill transfer; to date, however, no studies have examined how expert facilitators can transfer implementation facilitation skills to healthcare system change agents. This qualitative descriptive study begins to address this gap by identifying twenty-one techniques in a novel model for transferring skills. Twenty of these techniques, identified in mentoring, coaching, and/or cognitive apprenticeship literature, have been utilized to transfer other complex skills. We identified an additional technique, using comparisons to clinical skills and activities, in this study.

The process of transferring skills to develop expertise in implementation facilitation is, not surprisingly, multifaceted. Not only do facilitators need a wide variety of skills [11, 65], these skills are both complex and overlapping [19]. Facilitators must be able to (1) assess the needs and resources of organizations and its stakeholders [66], (2) identify barriers and enablers that will hinder or support the implementation process [1], (3) select appropriate implementation strategies and tailor them to the setting [67], (4) respond to changes in environment [68], and (5) intervene in the appropriate organizational level [11]. According to i-PARIHS framework developers, facilitators also have to respond to the unpredictable and constantly changing relationship between the characteristics of the innovation, recipients of the innovation, and layers of organizational context [1, 18]. Although there are explicit dimensions to implementation facilitation knowledge, e.g., someone with facilitation expertise can provide detailed descriptions of activities facilitators perform, there are also tacit dimensions to this knowledge, embedded in experience [66], e.g., the metacognitive strategies experts use for selecting activities likely to result in successful outcomes.

Nonaka’s Organizational Knowledge Creation theory provides insight into how the various techniques applied by the expert supported the transfer of explicit and tacit dimensions of implementation facilitation knowledge and skills. Specifically, Nonaka suggests that knowledge is created through the conversion of tacit knowledge to tacit and explicit knowledge and through the conversion of explicit knowledge to both explicit and tacit knowledge [69]. Mechanisms needed for conversion are different for these modes. For example, an expert’s tacit knowledge is converted to a novice’s tacit knowledge through observation; thus, *modeling* is a core technique for accomplishing this conversion. The conversion of tacit knowledge to explicit knowledge, a process Nonaka calls externalization, is often done using metaphors and analogies. Thus, *sharing experiences and telling stories*, *using comparisons to clinical skills*, and *using heuristics* are techniques useful for transferring expert tacit knowledge to explicit knowledge for the learner. *Making thinking visible* is also a way of externalizing tacit knowledge. In Nonaka’s model, explicit knowledge can be converted to tacit knowledge by internalization. The technique of *coaching* supports this process. As learners do what experts suggest and then receive feedback on their performances, experts’ explicit knowledge is converted to learners’ tacit knowledge. Finally, the conversion of explicit knowledge to explicit knowledge is accomplished through a process of exchanging and combining knowledge between individuals. The two participatory techniques identified in this study, *working together* and *providing consultation*, support the creation of knowledge by combining the explicit knowledge of the expert and the learner. To apply these transfer techniques, experts will need to consider which of them will best support trainees’ learning. For example, if trainees are novice to implementation facilitation, experts will likely need to provide them with information (*teaching*) and *model* how to conduct implementation facilitation activities. To support trainees’ learning, the expert will need to select techniques that can help trainees understand, apply, and generalize implementation facilitation processes (*cognitive supports*); build and enhance trainees’ sense of competence, identity as facilitators, and effectiveness (*psychosocial supports*); support their assessment, planning, and learning skills (by *promoting self-learning*); and provide them with opportunities to learn skills (*structural supports*).

Our exploratory study also found patterns in the expert’s interactions with facilitation trainees and site stakeholders that supported the transfer of skills. Such patterns are typical of apprenticeship relationships. For example, in medical residency and training programs, there is initially a higher intensity of interaction between mentors and residents. As the resident experiences more and diverse situations, responds appropriately, and exhibits competence, the mentor pulls back; and the resident practices with increasing independence. The similarity of the medical apprenticeship model [70] to the patterns of interaction we found is not surprising; the expert was a physician and was likely influenced by the way she had been trained to develop complex skills. It is possible that expert facilitators with different training and backgrounds might interact with trainees differently. Future studies should explore additional ways that the relationship between experts and trainees can support the skills transfer process.

Scholars have concluded that tailored support and education are needed to develop facilitation skills [23, 43, 44]; however, literature is silent about how expert facilitators tailor their efforts. Although other literature streams suggest that mentoring activities vary with mentee needs and organizational context [49, 71], they also do not provide us with information about the tailoring process. In this exploratory study, we assigned level of focus ratings to the expert’s efforts to transfer particular skills, compared ratings for each trainee, and explored qualitative data for potential reasons for variation between trainees. We found that the expert tailored her level of focus on particular implementation facilitation skills to trainees’ characteristics and skills, as well as the organizational context. The latter is consistent with research suggesting that facilitators and others providing implementation support, e.g., knowledge brokers, tailor what they do to organizational context [45, 72–74]; and that the characteristics of facilitators may influence implementation success [75]. Future work should explore the tailoring process further, including how experts adapt the skills transfer content (i.e., particular skills) and process (i.e., their level of focus on particular skills and the methods and techniques they use to transfer those skills), as well as what other issues experts need to consider when transferring facilitation skills.

There were several characteristics of this study that may affect the transferability of findings to other efforts to help novice or less experienced facilitators develop the skills they need to support implementation. First, this was an exploratory study of the methods one expert facilitator utilized to transfer skills to two initially novice facilitators. Additionally, facilitators focused on supporting implementation of a particular complex evidence-based program, PCMHI, in healthcare settings that would have been unable to implement PCMHI on their own. A larger study and/or one implementing a different innovation might reveal additional skills transfer processes.

Second, at the time of the study, we had no training materials and did not offer formal training opportunities. Thus, the skills transfer process was conducted during interactions between the expert, the facilitation trainees, and site stakeholders. Providing foundational information about implementation facilitation early in the training process would likely have impacted our study findings. We have since developed materials, in part based on findings from this study, and now provide training for studies and initiatives applying implementation facilitation [76]. Future research should explore how initial intensive training, in addition to ongoing support, impacts the transfer process.

Finally, the facilitation strategy was an intensive strategy informed by implementation science and designed to address all barriers that facilitators encountered and maximize the potential for implementation success. There are many approaches to implementation facilitation. Facilitators in this study may have needed a broader range of skills than facilitators who are using a more limited approach. Thus, the range of techniques and processes experts use to transfer facilitation skills may vary based on characteristics of the facilitation strategy. However, because the expert applied all of the techniques identified in mentoring, coaching, and cognitive apprenticeship literature, our model provides others with a comprehensive selection of techniques for transferring implementation facilitation skills.

## Conclusions

This exploratory study viewed the journey from novice to expert facilitator through the lens of the expert who transferred the skills needed to support implementation of an evidence-based program. As the first study to document the process of transferring implementation facilitation skills, its findings may have research and practical implications. Findings lay the foundation for future research to further explore the transfer process; how this transfer process varies across types of experts, settings, innovations, and characteristics of trainees; and whether there are techniques and processes that are consistent across all expert/trainee dyads and thus “core” to the skills transfer process. Study findings may also have practical implications for those planning implementation facilitation programs or helping others develop these skills. In addition to facilitation skills, experts need to know how to facilitate learning. Study findings propose twenty-one techniques experts can apply when transferring implementation facilitation skills to others. Although findings are exploratory, they confirm that experts also need to consider how they might adapt their emphasis on particular skills to the characteristics, existing skills, and needs of those they are training. Additionally, findings may be helpful to new facilitators who lack training opportunities. Understanding what we have learned about how skills are transferred may suggest options for seeking the support they need.

Finally, the development and growth of implementation science have been a response to the significant gap between evidence for clinical innovations and its implementation into clinical practice. There is now a parallel gap between implementation science knowledge and its routine application in healthcare organizations [77]. In this study, an expert transferred implementation facilitation skills to internal healthcare system change agents, thereby building capacity for future implementation efforts. Our findings contribute new knowledge about how experts can embed the skills needed to apply implementation strategies and thus improve implementation of evidence-based practice and policy in healthcare organizations.

## Supplementary Information

**Additional file 1:.** Skills transfer techniques and processes identified in mentoring, coaching, and cognitive apprenticeship literature. Description: This file contains a table of the skills transfer techniques and processes found in mentoring, coaching, and cognitive apprenticeship literature which informed the development of the qualitative code list applied in the study.

## Data Availability

The data generated by interviews with facilitators during this study are not publicly available due to the potential for compromising the privacy of study sites and their staff.

## Abbreviations

EF: Expert facilitator
FT: Facilitation trainee
i-PARIHS: Integrated-Promoting Action on Research Implementation in Health Services
PCMHI: Primary care mental health integration
VA: Department of Veterans Affairs
