# Supplementary material for: From novice to expert: methods for transferring implementation facilitation skills to improve healthcare delivery
Source: Implement Sci Commun. 2021 Apr 8;2:39. doi: 10.1186/s43058-021-00138-5 (PMC8033694; doi:10.1186/s43058-021-00138-5)
Supplement: Supplementary file 1 — Additional file 1:. Skills transfer techniques and processes identified in mentoring, coaching, and cognitive apprenticeship literature. Description: This file contains a table of the skills transfer techniques and processes found in mentoring, coaching, and cognitive apprenticeship literature which informed the development of the qualitative code list applied in the study. [file 43058_2021_138_MOESM1_ESM.docx]

**Additional File 1**

**Skills transfer techniques and processes identified in mentoring, coaching and cognitive apprenticeship literature**

|  |  | **Literature Streams** | | |
| --- | --- | --- | --- | --- |
| **Techniques and processes** | | **Mentoring^*^** | **Coaching^**^** | **Cognitive Apprenticeship** |
| Co-teaching/problem-solving, etc. | | [1] | [2] | [3-5] |
| Consultation | | [1] |  | [5] |
| Encouraging articulation | |  | [6] | [4, 5, 7, 8] |
| Encouraging exploration | |  | [9] | [3-5, 8] |
| Encouraging reflection | | [1, 10] | [9] | [4, 5, 7, 11] |
| Facilitating exposure and visibility | | [10, 12-14] |  |  |
| Fading or separation | | [15, 16] |  | [3, 4] |
| Giving challenging assignments | | [12-14] |  | [4] |
| Making thinking visible | | [17] |  | [18] |
| Modeling | | [1, 10, 12-14, 19, 20] | [2, 21] | [4, 5, 7, 18] |
| Observation and assessment | | [1, 12, 22] | [6, 9] | [3, 4, 18] |
| Promoting interests | | [13, 23] |  |  |
| Providing acceptance, confirmation and support | | [6, 10, 13, 14, 24] | [25] | [7] |
| Providing feedback | | [10, 13, 19] | [21, 26] | [4, 5] |
| Providing protection | | [13, 14] |  | [18] |
| Providing suggestions, advice, reminders | | [1, 10, 12-14, 19, 20, 22, 24] | [21, 25, 27] | [3-5] |
| Scaffolding | |  |  | [3, 4, 7] |
| Sequencing | | [1, 10] |  | [4] |
| Sharing experiences and telling stories | | [28] |  | [7] |
| Stepping in and out | | [1] |  | [4, 11] |
| Teaching/instructing | | [10, 19] | [25] | [4] |
| Utilizing heuristics | |  |  | [4, 7] |
| ^*^Techniques and processes vary across different types of mentoring literature, e.g., stepping and stepping out is only found in educative mentoring literature. **Although empirical literature on coaching is small, techniques also vary across different types of coaching literature. | | | | |

References

1. Schwille SA. The professional practice of mentoring. Am J Educ*.* 2008;115(1):139-67. <https://doi.org/10.1086/590678>.

2. Kretlow AG, Cooke NL, Wood CL. Using in-service and coaching to increase teachers’ accurate use of research-based strategies. Remedial Spec Educ*.* 2012;33(6):348-61. <https://doi.org/10.1177%2F0741932510395397>.

3. Browne DL, Ritchie DC. Cognitive apprenticeship: a model of staff development for implementing technology in schools. Contemp Educ*.* 1991;63(1):28.

4. Collins A, Brown JS, Holum A. Cognitive apprenticeship: making thinking visible. Am Educ*.* 1991;15(3):6-11.

5. Feinstein RE, Huhn R, Yager J. Apprenticeship model of psychotherapy training and supervision: utilizing six tools of experiential learning. Acad Psychiatry*.* 2015;39(5):585-9. <https://doi.org/10.1007/s40596-015-0280-6>.

6. Watt L. Mentoring and coaching in the workplace. Canadian Manager*.* 2004;29(3):14-6.

7. Nickle P. Cognitive apprenticeship: laying the groundwork for mentoring registered nurses in the intensive care unit. Dynamics*.* 2007;18(4):19-27.

8. Stalmeijer RE, Dolmans DH, Wolfhagen IH, Muijtjens AM, Scherpbier AJ. The Maastricht Clinical Teaching Questionnaire (MCTQ) as a valid and reliable instrument for the evaluation of clinical teachers. Acad Med*.* 2010;85(11):1732-8. <https://doi.org/10.1097/ACM.0b013e3181f554d6>.

9. Passmore J, Fillery-Travis A. A critical review of executive coaching research: a decade of progress and what's to come. Coaching: An International Journal of Theory, Research and Practice*.* 2011;4(2):70-88. <https://doi.org/10.1080/17521882.2011.596484>.

10. Ali PA. Professional development and the role of mentorship. Nurs Stand*.* 2008;22(42).

11. Cope P, Cuthbertson P, Stoddart B. Situated learning in the practice placement. J Adv Nurs*.* 2000;31(4):850-6. <https://doi.org/10.1046/j.1365-2648.2000.01343.x>.

12. Bouquillon EA, Sosik JJ, Lee D. ‘It’s only a phase’: examining trust, identification and mentoring functions received across the mentoring phases. Mentor Tutoring*.* 2005;13(2):239-58. <https://doi.org/10.1080/13611260500105808>.

13. Kram KE, Isabella LA. Mentoring alternatives: the role of peer relationships in career development. Acad Manage J*.* 1985;28(1):110-32.

14. Wanberg CR, Welsh ET, Hezlett SA. Mentoring research: a review and dynamic process model. Res Pers Hum Res Man*.* 2003;22:39-124. <https://doi.org/10.1016/S0742-7301(03)22002-8>.

15. Mills J, Lennon D, Francis K. Mentoring matters: developing rural nurses knowledge and skills. Collegian*.* 2006;13(3):32-6. <https://doi.org/10.1016/S1322-7696(08)60530-2>.

16. Sambunjak D, Straus SE, Marusic A. A systematic review of qualitative research on the meaning and characteristics of mentoring in academic medicine. J Gen Intern Med*.* 2010;25(1):72-8. <https://doi.org/10.1007/s11606-009-1165-8>.

17. Ness V, Duffy K, McCallum J, Price L. Supporting and mentoring nursing students in practice. Nurs Stand*.* 2010;25(1).

18. Ding H. The use of cognitive and social apprenticeship to teach a disciplinary genre: initiation of graduate students into NIH grant writing. Writ Commun*.* 2008;25(1):3-52. <https://doi.org/10.1177%2F0741088307308660>.

19. Butler MR, Felts J. Tool kit for the staff mentor: strategies for improving retention. J Contin Educ Nurs*.* 2006;37(5):210-3. <https://doi.org/10.3928/00220124-20060901-10>.

20. Roberts A. Mentoring revisited: a phenomenological reading of the literature. Mentor Tutoring*.* 2000;8(2):145-70. <https://doi.org/10.1080/713685524>.

21. Geist LJ, Cohen MB. Commentary: Mentoring the mentor: executive coaching for clinical departmental executive officers. Acad Med*.* 2010;85(1):23-5. <https://doi.org/10.1097/ACM.0b013e3181c46c5a>.

22. Hudson P, Skamp K, Brooks L. Development of an instrument: mentoring for effective primary science teaching. Sci Educ*.* 2005;89(4):657-74. <https://doi.org/10.1002/sce.20025>.

23. Clutterbuck D. The international perspective on mentoring. In: Ragins B, Kram K, editors. The handbook of mentoring at work: theory, research and practice Thousand Oaks, CA: Sage Publications; 2007. p. 633-56.

24. Hunt DM, Michael C. Mentorship: A career training and development tool. Acad Manage Rev*.* 1983;8(3):475-85.

25. Fixsen DL, Naoom S, Blase KA, Friedman RM, Wallace F. Implementation research: a synthesis of the literature. Tampa, FL: University of South Florida, Louis de la Parte Florida Mental Health Institute, 2005.

26. Feldman DC, Lankau MJ. Executive coaching: a review and agenda for future research. J Manage*.* 2005;31(6):829-48. <https://doi.org/10.1177%2F0149206305279599>.

27. Fielden SL, Davidson MJ, Sutherland VJ. Innovations in coaching and mentoring: implications for nurse leadership development. Health Serv Manage Rev*.* 2009;22(2):92-9. <https://doi.org/10.1258/hsmr.2008.008021>.

28. Lankau MJ, Scandura TA. Mentoring is a forum for personal learning in organizations. In: Ragins B, Kram KE, editors. The handbook of mentoring at work: theory, research, and practice. Thousand Oaks, CA: Sage Publications; 2007. p. 95-122.
